# Maternal Parenting Strategies and Daughters’ Perceived Weight Teasing as Predictors of Restrictive Eating Behaviors

**DOI:** 10.3390/nu18162717

**Published:** 2026-08-20

**Authors:** Norma Olvera, Molly R. Matthews-Ewald, Tamal J. Roy, Rhonda Scherer, Weihua Fan, Consuelo Arbona

**Affiliations:** 1 Department of Psychological, Health & Learning Sciences, University of Houston 3623 Cullen Blvd., McElhinney Hall, Room 3001, Houston, TX 77204, USA; tjroy@cougarnet.uh.edu (T.J.R.); rlschere@central.uh.edu (R.S.); wfan@central.uh.edu (W.F.); consueloa@central.uh.edu (C.A.); 2 WhitworthKee Consulting, LLC, Washington, DC 20003, USA; mmatthewsewald@whitworthkee.com

**Keywords:** Hispanic, mothers, pre-adolescent girls, restrictive behaviors, weight teasing, obesity

## Abstract

**Background/Objective**: Restrictive eating behaviors during early adolescence may increase the risk of disordered eating, yet little is known about modifiable family and social factors influencing these behaviors. This study investigated associations between maternal parenting strategies, weight teasing, and restrictive eating behaviors among 168 Hispanic mother–daughter dyads. **Methods**: Mothers and daughters independently completed surveys assessing demographic and acculturation characteristics, and their height and weight were objectively measured. Mothers also completed the parenting strategies measures, while daughters completed the weight teasing and restrictive eating behaviors measures. **Results**: Descriptive analyses indicated that 66.1% of mothers and 98.9% of daughters were classified in either the overweight or obese categories. Hierarchical multiple regression analyses showed that greater use of maternal limit-setting strategies was associated with lower levels of restrictive eating behaviors in daughters (*β* = −0.213, *p* = 0.017), whereas more frequently perceived weight teasing was associated with greater restrictive eating behaviors (*β* = 0.404, *p* < 0.001). The final model was statistically significant, *F*(8, 126) = 5.139, *p* < 0.001, explaining 19.8% of the variance in daughters’ restrictive eating behaviors. **Conclusions**: These findings suggest that interventions that strengthen supportive parenting strategies and reduce weight stigma may help prevent restrictive eating behavior in this population.

## 1. Introduction

Restrictive eating behaviors among adolescent girls represent a significant public health concern due to their association with disordered eating, nutritional inadequacies, and adverse psychological consequences [1]. Restrictive eating encompasses a range of behaviors characterized by the intentional limitation of food intake for the purpose of controlling weight or body shape, often driven by concerns regarding body image and sociocultural ideals of body thinness [2]. Considered an early manifestation of eating pathology, restrictive eating has been associated with increased risk for subsequent eating disorder symptoms during adolescence [3]. For instance, restrictive eating behaviors may represent early warning signs of anorexia nervosa. Thus, identifying these restrictive eating behaviors during adolescence may facilitate earlier recognition and intervention among youth at risk [4,5]. In addition, nutritional deficiencies and impaired psychosocial functioning are well-recognized consequences of clinically significant restrictive eating disorders [6]. Research consistently demonstrates that adolescent girls are disproportionately affected by restrictive eating behaviors compared to boys [2,7], highlighting the importance of identifying factors that may contribute to the emergence and maintenance of these behaviors in early adolescent girls.

Among contributing factors, maternal characteristics and parenting practices have received considerable attention, especially among mothers of young children [3]. Mothers often serve as primary caregivers and influential socialization agents who shape children’s attitudes toward food, body image, and weight management [8,9]. Through their parenting practices, behavioral modeling, and weight-related communication, mothers both directly and indirectly communicate beliefs about food, body weight, health, and appearance that shape how daughters perceive food and regulate their own eating behaviors [10,11]. Maternal body mass index (BMI) has also been consistently associated with children’s eating behaviors and weight-related outcomes [10]. Children of mothers with higher BMI are more likely to develop overweight or obesity, a relationship that reflects the combined influence of inherited genetic susceptibility as well as shared prenatal, environmental, and behavioral factors [10,12]. Relatedly, mothers’ perceptions and concerns regarding their children’s weight are associated with greater use of controlling feeding practices, including restriction and monitoring [13]. Because children of mothers with higher BMI are at increased risk for overweight or obesity [14,15], maternal concerns regarding their daughters’ weight may become more salient and contribute to greater use of parenting practices intended to regulate food intake within the household [13]. Thus, maternal BMI may serve as an important contextual factor influencing the development of parenting strategies that communicate messages about eating.

Maternal feeding strategies represent another important family-level influence on children’s eating behaviors. Feeding practices are the goal-directed strategies that parents use to influence what, when, and how much their children eat [16,17]. These practices have been consistently associated with children’s eating behaviors and appetite regulation across childhood, including among Hispanic families [3,18]. Three parenting practices frequently studied in the literature include monitoring, control, and structured (also referred to as limit-setting) feeding. Monitoring refers to parents’ awareness and supervision of their children’s consumption of specific foods, particularly foods considered less healthful [19]. This practice involves observing and tracking children’s dietary intake without necessarily imposing restrictions. Research examining monitoring has produced mixed findings. Parental monitoring has generally been associated with healthier dietary behaviors by increasing parents’ awareness of children’s food intake and providing opportunities to guide food choices [20,21]. However, other studies have found that parental monitoring is associated with less favorable outcomes, including increased child weight concern, greater dietary restraint, and higher risk for disordered eating attitudes. These inconsistent findings may reflect developmental differences in adolescents’ growing need for autonomy, as monitoring practices may be experienced differently depending on whether they are perceived as autonomy-supportive guidance or psychologically controlling [22]. When parental monitoring is perceived as emphasizing weight or appearance rather than health, it may contribute to greater dietary restraint and disordered eating attitudes instead of fostering adaptive eating behaviors [23,24]. However, when parenting practices are experienced as controlling rather than supportive, they may undermine children’s ability to self-regulate eating and are associated with less adaptive eating behaviors [23].

Controlling parenting practices include regulating the amount and type of food a child is allowed to consume. Although parents often use restriction to prevent excessive weight gain and/or promote healthy eating, highly controlling feeding practices have been associated with poorer eating self-regulation, including reduced responsiveness to internal hunger and satiety cues and greater eating in the absence of hunger [25]. Restrictive feeding practices can increase children’s preoccupation with restricted foods, contribute to dysregulated eating behaviors, and foster less adaptive relationships with food [25,26]. Restrictive feeding practices may communicate parental concerns regarding body weight and eating, potentially increasing adolescents’ engagement in dietary restraint and other disordered eating behaviors [3,24]. Importantly, controlling parenting practices, in general, appear to be associated with disordered eating behaviors in youth [27].

In contrast to control, limit-setting feeding strategies emphasize consistent and appropriate boundaries, through which children develop autonomy around eating within a structured but non-coercive environment [3,16,28]. Emerging evidence suggests that structured (limit-setting) feeding environments promote healthier, self-regulated eating behaviors in children [29,30]. Unlike coercive feeding practices, limit-setting may promote healthier eating by fostering a predictable food environment that supports children’s internal regulation and autonomy around eating. Consequently, these structured feeding practices may be less likely to contribute to dietary restraint than externally controlling approaches [16,28,30]. Nevertheless, little research has examined whether maternal limit-setting practices are associated with restrictive eating among adolescent girls, particularly among Hispanic populations, when considered alongside other maternal feeding practices such as monitoring and control.

Weight teasing, defined as criticism, ridicule, or negative comments directed toward an individual’s body weight or body shape, has been consistently associated with adverse psychological and behavioral outcomes, including body dissatisfaction, depressive symptoms, unhealthy weight-control behaviors, and disordered eating pathology [31,32,33,34]. Although weight teasing occurs across multiple social contexts (e.g., peers, parents, siblings, teachers, or coaches), family-based weight teasing may be particularly influential because it originates within the home environment, where parents play a vital role in shaping children’s eating behaviors and body image [33,35]. Weight teasing by parents and peers has been consistently associated with disordered eating behaviors, including binge eating, emotional eating, and other unhealthy weight-control behaviors among adolescents [32,36]. Collectively, maternal BMI, maternal parenting practices, and weight teasing may represent important risk factors for disordered eating behaviors such as restrictive eating. Mothers’ concerns regarding weight may influence both parenting behaviors and weight-related communication, creating a family environment that emphasizes weight control. Exposure to multiple weight-focused messages within the home may increase adolescents’ engagement in restrictive eating behaviors. However, relatively little is known about the association between parenting styles and disordered eating behaviors of their children [27].

One theoretical perspective for understanding how maternal BMI, parenting practices, and weight teasing may influence daughters’ restrictive eating behaviors is the Sociocultural Theory of body image and eating disorders [37,38]. According to this theory, societal appearance ideals are transmitted through influential socialization agents, including family members, peers, and media [37,39]. Within the family context, mothers may serve as particularly influential socialization agents by modeling body image attitudes and eating behaviors and by communicating beliefs regarding weight, body shape, and dieting [40]. These messages transmitted within the family context may contribute to adolescents’ vulnerability to weight stigma, which has been associated with dietary restraint, unhealthy weight-control behaviors, binge eating, and other forms of disordered eating [8,9,40]. Maternal concerns about weight, weight-related communication, and parenting practices may communicate explicit or implicit messages regarding the importance of thinness and weight control to daughters [10,33]. When daughters internalize these messages, they may be more likely to engage in restrictive eating behaviors to conform to perceived standards of thinness [39,41]. Consistent with this framework, mothers who express greater concern about their children’s weight are more likely to engage in monitoring and controlling parenting practices [13,42], which may in turn reinforce weight-focused messages within the home environment. Beyond parenting practices, weight teasing from family members or peers may further amplify body dissatisfaction and internalization of thinness ideals, increasing daughters’ vulnerability to restrictive eating behaviors [36,43]. Together, these experiences may converge to communicate the importance of weight control and encourage daughters to adopt restrictive eating behaviors [3,24]. Sociocultural Theory thus provides a useful lens for understanding how maternal BMI, parenting practices, and weight-related social experiences may jointly shape daughters’ engagement in restrictive eating, particularly during the sensitive developmental period of early adolescence.

Despite growing evidence that parental factors influence adolescents’ eating behaviors, several important gaps remain in the literature. First, few studies have simultaneously examined maternal BMI, weight teasing, and multiple maternal parenting practices as predictors of restrictive eating behaviors among Hispanic mother–daughter dyads. Existing research has typically investigated these factors independently or has focused largely on non-Hispanic White populations, limiting understanding of how they may operate collectively within Hispanic families [44]. This gap is particularly important because Hispanic mothers and daughters may be exposed to competing cultural appearance ideals, including traditional Latino preferences for curvier body shapes alongside mainstream U.S. ideals that emphasize thinness [45,46]. Second, much of the existing literature has emphasized childhood overweight, obesity, or general eating behaviors rather than restrictive eating specifically, despite evidence that restrictive eating represents an early behavioral manifestation of eating pathology. Third, maternal strategies have been frequently studied as a broad construct, while few studies have examined the collective and unique contribution of parental control, monitoring, and limit-setting parenting practices to adolescents’ restrictive eating behaviors. Addressing these gaps may improve understanding of family mechanisms underlying restrictive eating and inform culturally responsive, family-based prevention strategies for Hispanic girls.

Thus, the present study investigated the associations among maternal feeding and physical activity strategies (e.g., control and limit setting), daughters’ perceived weight teasing, and daughters’ restrictive eating behaviors within a theoretically informed framework. Based on a sociocultural framework, the following two hypotheses were proposed: (1) maternal control and monitoring parenting strategies will be positively associated with daughters’ restrictive eating behaviors, whereas maternal limit setting strategy will be negatively associated with restrictive eating behaviors; and, (2) daughters’ perceived weight teasing will be positively related to restrictive eating behaviors above and beyond the effects of maternal parenting strategies.

## 2. Materials and Methods

### 2.1. Participants

The study sample included 168 Hispanic mother–daughter dyads drawn from different baseline cohorts enrolled in a summer healthy lifestyle intervention conducted between 2009–2012 and 2017–2019. Eligibility criteria included: (1) mother and daughter self-identifying as Hispanic or African American (only Hispanic dyads were included in the present analysis); (2) daughters aged 9–14 years; (3) daughters without medical or physical conditions that limited participation in physical activity; (4) daughters classified as having overweight or obesity. Maternal weight status was not an eligibility criterion; and (5) mother and daughter must reside in the same household. Participants were recruited through community-based social service agencies, healthcare clinics, school nurses, community referrals, and direct outreach by the research team. All study procedures were approved by the Institutional Review Board (IRB) of the University of Houston (protocol #05271, approval date 28 June 2009; and protocol #16447, approval date 17 June 2016) and written informed consent from mothers and assent from daughters were obtained prior to their participation in the study.

### 2.2. Procedures

Before data collection, we invited potential mother–daughter dyads to a group orientation session led by trained bilingual research assistants. During the session, study procedures, requirements, and expectations were explained, and participants had the opportunity to ask questions. At the end of the orientation, mothers provided written informed consent and parental permission, and daughters provided written assent. Consenting dyads were then scheduled for a 90 min pre-intervention assessment conducted in university classrooms and gymnasium facilities. At the beginning of the assessment session, mothers and daughters completed separate self-administered questionnaires in their preferred language (English or Spanish) to ensure independent responses. Bilingual research assistants were available to answer questions as needed. Overall, 40% of mothers and 79% of daughters completed the surveys in English. Following survey completion, trained bilingual research assistants measured participants’ height and weight using standardized protocols. The set of administered measures can be found in the accompanying Supplementary File S1: Study_Questionnaires.

### 2.3. Measures

#### 2.3.1. Demographic Survey

As noted, each mother and daughter independently completed a short demographic survey consisting of questions regarding age, birthplace, educational attainment, and self-described ethnicity. The maternal demographic survey included additional questions concerning their household income, family size, and occupation.

#### 2.3.2. Acculturation Survey

Mothers completed the 24-item Bidimensional Acculturation Scale (BAS) [47] which assessed two cultural domains (i.e., Hispanic and U.S. culture) based on three subscales: language use (e.g., How often do you speak/think in English?), linguistic proficiency (e.g., How well do you speak Spanish?), and electronic media usage (e.g., How often do you watch television in English?). Mothers answered questions on a 4-point Likert-type scale ranging from 1 (almost never/very poorly) to 4 (almost always/very well). An average score of ≥2.5 on the mainstream culture domain was indicative of a mother’s high acculturation to U.S. culture. Mothers with an average score < 2.5 on the U.S. domain were classified as “low acculturation to U.S. Culture”. The BAS measure is recognized as a valid and reliable measure of acculturation among Hispanics in the United States. In this study, the internal consistency of the BAS scores was excellent for both the Hispanic domain (Cronbach’s α = 0.93) and the U.S. culture domain (Cronbach’s α = 0.97).

Daughters completed the Short Acculturation Scale for Hispanic Youth (12 items) [48], which assessed acculturation to mainstream culture in terms of language use/proficiency (9 items; e.g., What language do you usually speak at home?) and social relations (3 items; e.g., Your close friends are) on a 5-point scale ranging from 1 (Only Spanish) to 5 (Only English) and from 1 (All Hispanic) to 5 (All Non-Hispanic), respectively. A score of >30 indicated high acculturation, and a score of <30 indicated low acculturation. The internal consistency of participants’ scale scores was good (Cronbach’s α = 0.82).

#### 2.3.3. Obesity Status

Trained research assistants measured maternal and daughter’s body weight (in kilograms) while wearing light clothes without shoes using a scale (Tanita SC-331S; Tanita Corporation, Tokyo, Japan) and rounded to the nearest 0.1 kg. Participants’ height was measured using the Seca 213 stadiometer (Seca GmbH & Co. KG, Hamburg, Germany) and rounded to the nearest 0.1 cm. Body mass index (BMI) was calculated by the standard formula (weight/height^2^), and BMI values were then used to identify the age- and gender-specific percentile for each daughter using the Centers for Disease Control and Prevention growth charts [49]. Based on these percentiles, each daughter was classified as either with overweight (>85th–94th percentile) or with obesity (≥95th percentile). Mothers’ weight status was also calculated using the World Health Organization (WHO) obesity classification. That is, mothers with a BMI < 24.9 kg/m^2^ were classified as being of a healthy weight; mothers with a BMI between 25.0 kg/m^2^ and 29.9 kg/m^2^ were classified as with overweight; and mothers with a BMI > 30 kg/m^2^ were classified as with obesity [50].

#### 2.3.4. Parenting Strategies for Eating and Physical Activity Scale (PEAS)

To assess parenting strategies for eating and physical activity, mothers completed the Parenting Strategies for Eating and Activity Scale [51]. This validated scale assesses several parenting strategies for eating and physical activity in youth: limit setting, monitoring, control, discipline, and reinforcement. Consistent with the literature, we focused on three parenting strategies for this study: (1) monitoring food consumption (3 items, e.g., How much do you keep track of the high fat food your child eats?), (2) control (5 items, e.g., My child should always eat all the food I give her.), and (3) limit setting for eating and screen time (6 items, e.g., I limit the amount of snacks my child eats. I limit the amount of time my child watches TV during the week). Response options for each of the strategies ranged from 1 never or disagree to 5 always or agree. In this study, the reliability of the PEAS subscales ranged from approaching acceptable to excellent. The control subscale demonstrated marginal internal consistency (Cronbach’s α = 0.66), falling slightly below the conventional threshold for satisfactory reliability, whereas the limit-setting (Cronbach’s α = 0.91) and monitoring (Cronbach’s α = 0.87) subscales showed excellent and acceptable internal consistency, respectively.

#### 2.3.5. Weight Teasing

Daughters’ weight teasing was assessed using 11 items drawn from the McKnight Risk Factor Survey IV-weight teasing scale (MRFS-IV) [52] as part of a broader assessment of risk factors for eating disorders. The weight teasing scale includes 11 items. Items 1–8 (weight teasing peers’ subscale) assessed teasing in the past year by boys and girls, attempts to change weight to avoid teasing, and the perceived social impact of teasing. For instance, items include “In the past year, how often have girls made fun of you because of your weight?” and “In the past year, how often have you tried to change your weight so you would not be teased by boys?” Items 9–11 assess negative weight-related comments from adults (mother, father, teacher/coach). Samples of items include: In the past year, how often has your father made a comment to you about your weight or your eating that made you feel bad? In the past year, how often has your mother made a comment to you about your weight or your eating that made you feel bad? In the past year, how often has a teacher or coach made a comment to you about your weight that made you feel bad? Some studies report a combined score of the eight peer teasing items plus three adult comment items and labeled this score negative comments about weight, with higher scores reflecting a greater frequency of negative comments about weight. In the current study, this measure demonstrated good internal consistency (Cronbach’s α = 0.85).

#### 2.3.6. Restrictive Eating Behaviors

Daughters’ engagement in restrictive eating behaviors was assessed using four items from the McKnight Risk Factor Survey-IV (MRFS-IV) [50]. These restrictive eating behavior items assessed the frequency of the following behaviors in the past month: dieting, fasting for a day or more, cutting back on food intake, and skipping meals. Items were averaged so that higher scores reflected more frequent restrictive eating behavior. In this study, this subscale demonstrated adequate internal consistency (Cronbach’s α = 0.75).

### 2.4. Statistical Analysis

Descriptive analyses were conducted to describe the characteristics of mothers and daughters, including age, place of birth, annual family income, weight status, and acculturation. A series of Pearson’s correlations were performed to examine the bivariate associations among key study variables, informing the hierarchical regression specification and the interpretation of the findings. Missing data were handled using listwise deletion. To test hypothesis 1, that daughters whose mothers have higher BMI and daughters who report more frequent weight teasing will exhibit higher levels of restrictive eating; a series of Pearson’s correlations was performed. To test both hypotheses, a three-step hierarchical multiple regression was conducted. At Step 1, daughters’ age, mothers’ age, daughters’ BMI, and mothers’ BMI were entered as control variables. At Step 2, maternal parenting practices related to eating and activity were entered, including (1) parental control, (2) limit setting, and (3) monitoring of child diet and physical activity. At Step 3, daughters’ perceived weight teasing was entered to determine whether weight teasing explained additional variance in daughters’ restrictive eating behaviors after accounting for age, BMI, and maternal parenting strategy variables. Statistical significance was set at *p* < 0.05. Before conducting the hierarchical regression, the tolerance and variance inflation factors (VIFs) for all predictors were examined. In the final model, tolerance values ranged from 0.653 to 0.939, and VIF values ranged from 1.065 to 1.533, indicating no evidence of problematic multicollinearity among the predictors [53]. To ensure that findings were consistent across the study periods (2009–2012 and 2017–2019), a sensitivity analysis was conducted which included a dichotomous cohort variable (2009–2012 vs. 2017–2019) as an additional covariate at Step 1 of the hierarchical regression, alongside daughters’ age, mothers’ age, daughters’ BMI, and mothers’ BMI, with Steps 2 and 3 (maternal parenting strategies and daughters’ perceived weight teasing, respectively) in the primary model.

## 3. Results

### 3.1. Sample Descriptive Characteristics

The sample consisted of 168 Hispanic mother–daughter dyads. As shown in Table 1, mothers’ mean age was 38.9 years (*SD* = 6.6), and daughters’ mean age was 11.5 years (*SD* = 1.4). Over half of the mothers reported an annual household family income of less than $30,000. Most mothers were born outside the U.S. (81.0%, *n* = 136), primarily born in Mexico (69.6%, *n* = 117); 39.9% (*n* = 67) reported high acculturation, whereas 49.4% (*n* = 83) reported low levels of acculturation. In contrast, 92.9% (*n* = 156) of daughters were born in the United States, and 79.2% (*n* = 133) reported high acculturation. Regarding weight status, among mothers, 11.3% (*n* = 19) were of normal weight, 26.8% (*n* = 45) of mothers had overweight, and 39.3% (*n* = 66) had obesity. Among daughters, 18.5% (*n* = 31) were overweight, and 80.4% (*n* = 135) had obesity.

### 3.2. Correlations Between Key Variables

Pearson’s correlations revealed a significant positive association between daughters’ perceived weight teasing and daughters’ engagement in restrictive eating (*r* = 0.405, *p* < 0.001). However, the bivariate correlation between mothers’ BMI and daughters’ restrictive eating was not significant (*r* = 0.057, *p* = 0.465). We also explored correlations between the other key variables before conducting hierarchical regression to answer hypotheses 1 and 2. Specifically, correlational analysis indicated that restrictive eating was positively associated with parental control (*r* = 0.163, *p* = 0.035) and weight teasing (*r* = 0.405, *p* < 0.001). Daughters’ age was positively correlated with maternal age (*r* = 0.204, *p* = 0.008) and daughters’ BMI (*r* = 0.275, *p* < 0.001) and negatively correlated with monitoring (*r* = −0.257, *p* < 0.001). Daughter BMI was positively associated with maternal BMI (*r* = 0.424, *p* < 0.001) and weight teasing (*r* = 0.204, *p* = 0.015) and negatively associated with monitoring (*r* = −0.171, *p* = 0.028). Maternal BMI was negatively correlated with limit setting (*r* = −0.203, *p* = 0.009). Finally, limit setting and monitoring were positively correlated with one another (*r* = 0.474, *p* < 0.001).

### 3.3. Associations of Maternal Strategies, Daughters’ Perceived Weight Teasing, and Daughters’ Restrictive Eating Behaviors

As noted, a three-step hierarchical multiple regression model was conducted to test hypotheses 1 and 2. After reviewing the significant correlations, we examined the unique contributions of the predictors on daughters’ restrictive eating behaviors. As shown in Table 2, in Step 1, daughters’ age, mothers’ age, daughters’ BMI, and mothers’ BMI were entered as control variables. This model was not statistically significant, *F*(4, 130) = 0.510, *p* = 0.731. In Step 2, three maternal strategies related to eating and activity were added to the model. The addition of these maternal strategies significantly improved model fit, Δ*R*^2^ = 0.081, Δ*F*(3, 127) = 3.780, *p* = 0.012. However, the overall model was not significant, *F*(7, 127) = 1.928, *p* = 0.070. In Step 3, daughters’ perceived weight teasing was added to the model. The final model was statistically significant, *F*(8, 126) = 5.139, *p* < 0.001, explaining about 20% of the variance in daughters’ restrictive eating behaviors, *R*^2^ = 0.246, adjusted *R*^2^ = 0.198. The addition of perceived weight teasing significantly improved model fit, Δ*R*^2^ = 0.150, *F*(1, 126) = 25.063, *p* < 0.001.

In the final model, daughters’ perceived weight teasing was positively associated with restrictive eating behaviors, *B* = 0.435, *SE* = 0.087, *β* = 0.404, *p* < 0.001, 95% CI [0.263, 0.606], indicating that daughters who reported more frequent weight teasing also reported higher levels of restrictive eating behaviors. Maternal limit setting was negatively associated with daughters’ restrictive eating behaviors, *B* = −0.158, *SE* = 0.065, *β* = −0.213, *p* = 0.017, 95% CI [−0.286, −0.029]. That is, daughters of mothers who more frequently set limits on foods their daughters consumed and on sedentary activities (like watching television) were less likely to engage in restrictive eating behaviors.

Sensitivity analyses indicated that cohort was not a significant predictor of daughters’ restrictive eating, accounting for essentially no variance in the model (*β* = −0.003, *t* = −0.038, *p* = 0.970), indicating no systematic difference in restrictive eating across recruitment periods once demographics were accounted for. The overall model fit was likewise comparable (*R*^2^ = 0.246, adjusted *R*^2^ = 0.192, *F*(9, 125) = 4.532, *p* < 0.001), as were the increments in variance explained by parenting strategies (Step 2: Δ*R*^2^ = 0.081, *p* = 0.013) and weight teasing (Step 3: Δ*R*^2^ = 0.148, *p* < 0.001). Together, these results indicate that the observed associations are robust across recruitment periods and are not attributable to systematic cohort differences.

## 4. Discussion

This study investigated associations among maternal parenting strategies (including control, monitoring, and limit setting) and daughters’ perceived weight teasing with daughters’ restrictive eating behaviors among Hispanic mothers and their early adolescent daughters with overweight or obesity. Based on a sociocultural framework, we tested two hypotheses. First, we expected that maternal strategies characterized by greater control and monitoring would be positively associated with daughters’ restrictive eating behaviors, whereas maternal limit setting would be negatively associated with daughters’ restrictive eating behaviors. This hypothesis was partially supported. Study findings revealed an inverse association between maternal limit-setting and daughters’ restrictive eating behaviors among Hispanic early adolescent daughters. However, neither maternal control nor monitoring emerged as statistically significant predictors in the final model.

These findings suggest that maternal limit-setting may serve as a protective factor against restrictive eating behaviors in daughters. Within the context of parenting theory, supportive limit-setting strategies reflect an authoritative parenting style that promotes children’s autonomy within a structured environment and has consistently been associated with healthier dietary behaviors, greater self-regulation, and positive psychosocial outcomes [16,23,28]. Supportive limit-setting strategies may encourage consistent eating routines, promote eating self-regulation, and decrease the likelihood of unhealthy weight-control behaviors by providing children with clear expectations while preserving child autonomy [3,17,23,30].

Our findings are consistent with previous studies demonstrating that positive food parenting practices, characterized by responsiveness, appropriate structure, and autonomy support, are associated with healthier eating behaviors and lower risk of disordered eating among adolescents [3,23]. Although much of the existing literature has focused on parental monitoring, modeling, or control strategies in young children [10,14], considerably less attention has been given to supportive limit-setting as a distinct food parenting practice in relation to restrictive eating behaviors. Moreover, studies examining these associations among Hispanic preadolescent girls remain scarce. Given the significant role of parents in shaping children’s eating environments, particularly during early adolescence, these findings extend previous research by suggesting that limit-setting parenting strategies may serve as protective factors against the development of maladaptive eating behaviors.

Consistent with the second hypothesis, weight teasing emerged as a significant and strong contributor to restrictive eating behaviors. Daughters who reported more frequently perceived weight teasing reported higher levels of restrictive eating behaviors than their peers who reported less frequent weight teasing, when accounting for demographic characteristics, weight status, and parenting strategies. This finding aligns with a growing body of evidence demonstrating that weight teasing, criticism, and stigmatizing comments from family members or peers are associated with unhealthy weight-control behaviors, body dissatisfaction, and eating disorder symptoms [33,34,36]. Weight-related comments may reinforce societal ideals regarding thinness and increase internalized weight stigma, prompting adolescents to engage in restrictive eating to alter their body size or avoid further criticism [33,36,40]. The magnitude of the association between perceived weight teasing and restrictive eating behaviors observed in the present study suggests that reducing weight stigma within family and social environments may represent a key component of eating disorder prevention efforts [33,38]. Previous interventions have primarily focused on improving nutrition knowledge and promoting healthy lifestyle behaviors; however, the findings from the present study support growing recommendations that obesity prevention and health promotion programs also explicitly address weight teasing, appearance-focused communication, and weight stigma [33,38].

Families managing pediatric overweight and obesity often receive messages emphasizing weight reduction, which may unintentionally increase adolescents’ vulnerability to unhealthy weight-control behaviors if not delivered within supportive, non-stigmatizing contexts [6,33]. These findings are particularly relevant for Hispanic families, a population disproportionately affected by childhood obesity and related health disparities [10,54]. Within Hispanic families, parenting practices play an important role in shaping children’s eating behaviors and weight-related outcomes, suggesting that family-based interventions may be particularly effective for promoting healthy eating behaviors while preventing disordered eating [3,11,18,23]. At the same time, cultural norms regarding body size, gender expectations, and family communication may shape both parenting practices and adolescents’ responses to perceived weight teasing. Future research should further explore culturally specific mechanisms, including the influence of concerns of body size and other cultural perceptions of body image and acculturation [55], to better understand how these factors shape restrictive eating behaviors in this population.

Several limitations should be considered when interpreting these findings. First, the cross-sectional design precludes conclusions regarding causality or temporal ordering. It is possible that daughters who engage in restrictive eating behaviors elicit greater parental concern or become more sensitive to perceived weight-related comments. Longitudinal studies are needed to clarify the temporal sequence of these relationships and identify developmental pathways linking parenting, weight stigma, and restrictive eating over time. Second, daughters’ restrictive eating behaviors and exposure to negative weight comments were assessed using self-report measures, which may be subject to recall bias or social desirability bias. Third, while the parental control subscale was included in the model due to the importance of this strategy in the literature, we acknowledge that this subscale’s scores did not meet an acceptable internal reliability criterion. This may have attenuated the observed associations between parental control and daughters’ restrictive eating, contributing to the marginal rather than significant findings for this predictor. Future studies should include measures of parental control with stronger psychometric properties, particularly those validated with Hispanic samples, to further examine this construct’s role in Hispanic adolescents’ eating behaviors. Further, the marginal positive trends observed for control and monitoring indicate that these latter two strategies may operate differently from limit-setting, and perhaps counterproductively, in relation to daughters’ restrictive eating behaviors. However, more research is needed to further understand these relationships.

Fourth, the study was limited to Hispanic daughters with overweight or obesity who were seeking to participate in a healthy lifestyle intervention. This limits the generalizability of the findings, as our results apply only to a specific population of treatment-seeking Hispanic girls with overweight or obesity. In addition, the study’s focus on Hispanic mother–daughter dyads limits the ability to examine the influence of fathers, siblings, or other peers who may also contribute to adolescents’ eating behaviors. Fifth, the study relied on data collected across two distinct time periods (2009–2012 and 2017–2019); although the sensitivity analysis indicated the pattern of findings did not differ across cohorts, the possibility of unmeasured variables that may have impacted the cohorts differently remains. Future studies should consider whether findings replicate across cohorts collected in different sociocultural contexts.

Despite these limitations, the present study has important implications for clinical practice and for developing family-based interventions for Hispanic families raising daughters with overweight or obesity. A focus on early intervention is particularly important, given that restrictive eating behaviors may represent an early form of disordered eating such as anorexia nervosa. Identifying factors associated with these behaviors during early adolescence may inform prevention efforts for subsequent development of more severe, clinical eating disorders. In a clinical setting, healthcare providers could assess not only adolescents’ weight status and eating behaviors but also family communication about weight and the specific parenting strategies used to manage eating and physical activity at home. Interventions should prioritize structure-based, autonomy-supportive feeding practices over more restrictive or surveillance-oriented approaches.

Findings also support family-based interventions that consider strengthening supportive parenting practices, including developmentally appropriate limit-setting around eating while discouraging weight-based teasing. Parent education could include guidance on communicating about food and body weight in ways that emphasize health and well-being rather than weight or appearance, as well as strategies for responding constructively to adolescents’ eating concerns. For Hispanic families, interventions may also benefit from culturally responsive content that considers family values, communication patterns, and acculturation experiences. Because strategies differ from the more directive, control-based feeding approaches that may be normative in some families, interventions should be culturally responsive and engage the broader family unit, rather than the mother–daughter dyad alone, reflecting the multigenerational structure of many Hispanic households.

## 5. Conclusions

These findings highlight the importance of the family and social environment in shaping eating behaviors during early adolescence and suggest that both supportive parenting practices and perceived weight teasing may represent modifiable targets for interventions aimed at preventing disordered eating among Hispanic early adolescents. Specifically, maternal limit-setting emerged as a protective factor, while perceived weight teasing was the strongest independent predictor of restrictive eating in the final regression model. Intervention studies are warranted to determine whether modifying these family and social factors can reduce the development of restrictive eating behaviors during preadolescence. The focus on Hispanic mothers and early adolescent daughters addresses an important gap in the literature, as this population has been underrepresented in studies examining family influences on restrictive eating despite experiencing elevated risk for obesity and related psychosocial challenges.

## Figures and Tables

**Table 1:** Sample Descriptive Characteristics (*n* = 168 Hispanic Mother–Daughter Dyads).

Variable	Daughters	Mothers
Age in years, M (SD)	11.5 (1.4)	38.9 (6.6)
Place of birth, *n* (%)		
U.S.	156 (92.9)	32 (19.0)
Mexico	11 (6.5)	117 (69.6)
Central America	1 (0.6)	18 (10.7)
Other	0 (0.0)	1 (0.6)
Missing	0	0
Annual household family income, *n* (%)		
≤$30,000	—	91 (65.9)
>$30,000	—	47 (34.1)
Missing	*—*	30
Acculturation, *n* (%)		
High	133 (88.7)	67 (44.7)
Low	17 (11.3)	83 (55.3)
Missing	18	18
Weight status, *n* (%)		
Healthy weight	0 (0.0)	19 (14.6)
Overweight	31 (18.7)	45 (34.6)
Obesity	135 (81.3)	66 (50.8)
Missing	2	38

Note. Percentages are calculated based on participants with valid data for each variable; missing cases are reported as counts but are excluded from the denominator used to calculate percentages. Valid sample sizes were as follows: daughters: place of birth *n* = 168, acculturation *n* = 150, weight status *n* = 166; mothers: place of birth *n* = 168, income *n* = 138, acculturation *n* = 150, weight status *n* = 130. Daughters’ weight status was classified using CDC age- and sex-specific BMI percentiles, whereas mothers’ weight status was classified using WHO adult BMI categories. Because study eligibility criteria required daughters to be overweight or obese, no daughters were classified as normal weight.

**Table 2:** Hierarchical Regression Analysis Predicting Restrictive Eating Behaviors Among Hispanic Early Adolescent Daughters.

Variable	*B*	*SE*	95% CI	*β*	*t*	*p*
Model 1						
Daughter age	−0.035	0.053	[−0.140, 0.070]	−0.063	−0.65	0.516
Mother age	−0.003	0.011	[−0.025, 0.019]	−0.023	−0.26	0.794
Daughter BMI	−0.007	0.015	[−0.037, 0.023]	−0.048	−0.46	0.646
Mother BMI	0.010	0.011	[−0.012, 0.032]	0.093	0.92	0.361
Model 2						
Daughter age	−0.027	0.054	[−0.134, 0.080]	−0.048	−0.49	0.623
Mother age	−0.002	0.011	[−0.024, 0.020]	−0.013	−0.14	0.887
Daughter BMI	−0.001	0.015	[−0.031, 0.029]	−0.007	−0.07	0.944
Mother BMI	0.008	0.011	[−0.014, 0.030]	0.069	0.70	0.488
Control	0.145	0.062	[0.022, 0.268]	0.200	2.32	0.022
Limit setting	−0.145	0.071	[−0.285, −0.005]	−0.196	−2.04	0.043
Monitoring	0.211	0.102	[0.009, 0.413]	0.206	2.08	0.040
Model 3						
Daughter age	−0.021	0.049	[−0.118, 0.076]	−0.039	−0.43	0.669
Mother age	−0.002	0.010	[−0.022, 0.018]	−0.015	−0.19	0.853
Daughter BMI	−0.015	0.014	[−0.043, 0.013]	−0.102	−1.07	0.289
Mother BMI	0.006	0.010	[−0.014, 0.026]	0.057	0.63	0.533
Control	0.108	0.058	[−0.007, 0.223]	0.149	1.87	0.064
Limit setting	−0.158	0.065	[−0.286, −0.029]	−0.213	−2.42	0.017
Monitoring	0.156	0.094	[−0.030, 0.342]	0.152	1.66	0.099
Weight teasing	0.435	0.087	[0.263, 0.606]	0.404	5.01	<0.001

Note. *n* = 135 dyads with complete data on all model variables of 168 enrolled mother–daughter dyads; listwise deletion. *B* = unstandardized regression coefficient; *SE* = standard error of B; CI = confidence interval for *B*; *β* = standardized regression coefficient. BMI = body mass index.

## Data Availability

The data presented in this study are available upon request from the corresponding author after five years upon completion of the grant. The data are not publicly available due to concerns regarding privacy.

## Supplementary Materials

The following supporting information can be downloaded at: https://www.mdpi.com/article/10.3390/nu18162717/s1, File S1: Study_Questionnaires.
