# Supplementary material for: Maternal Parenting Strategies and Daughters’ Perceived Weight Teasing as Predictors of Restrictive Eating Behaviors
Source: Nutrients. 2026 Aug 20;18(16):2717. doi: 10.3390/nu18162717 (PMC13516391; doi:10.3390/nu18162717)
Supplement: Supplementary file 1 [file nutrients-18-02717-s001.zip › nutrients-4480787-supplementary.pdf]

CHILD MEASURES

BOUNCE
to build healthy families

Clinical and Physical Fitness Measures (Child)

ID: ________________________________ **Date:** ______________________________

Race/Ethnicity (circle one): White Black/African American Hispanic Other ____________

Gender (circle one): Female Male (Circle) PRE POST Other ___________

"What is your height?": _______________ **"What is your weight?":** _______________

Variable	Result		Average	Initials
**Today's Date** (mm/dd/yyyy)				
**DOB** (mm/dd/yyyy)				
**Age** (yrs)				
**Body Ht** (ft' in" i.e. 5' 2") (in and cm)	Trial 1<br>_____ ' _________ "<br>in: _________<br>cm: _________	Trial 2<br>_____ ' _________ "<br>in: _________<br>cm: _________	_____ ' _________ "<br>in: _________<br>cm: _________	
**Body Wt** (lbs and kg)	Trial 1<br>lb: _________<br>kg: _________	Trial 2		
**Body Fat** (%) (BIA)	Trial 1<br>____________	Trial 2	____________	
**Waist Circumference** (cm)	Trial 1<br>____________	Trial 2<br>____________	____________	
**Heart Rate** (bpm)	Trial 1<br>_________ X 2	Trial 2<br>_________ X 2	_________ X 2	
**Blood Pressure** (mmHg)	Trial 1<br>systolic: ________<br>diastolic: ________	Trial 2<br>systolic: ________<br>diastolic: ________	systolic: ________<br>diastolic: ________	
**1-mile Walk** (minutes)	Trial 1<br>1 Mile Time in minutes: __________			
**BMI** (To be calculated after measurement.)	BMI = $\frac{\text{Weight (kg)}}{(\text{Height(m)})^2}$ = __________ =			
**BMI Percentile** (To be calculated after measurement.)	__________________			

Date Entered: ________________________________ Initials: ________________

Date Double-checked: _________________________ Initials: ________________

BOUNCE
to build healthy families

Demographics and Technology (Child)

Name/ ID: ____________________ **Date:** ______________

1. **When is your birthday?** _____________ month _______________ day ______________ year

   Age: _______ years

2. **What grade are you in (currently)?** ☐ 3rd ☐ 4th ☐ 5th ☐ 6th ☐ 7th ☐ 8th ☐ 9th

3. **What is your ethnicity?** ☐ Hispanic (1) ☐ Black (2) ☐ White (3)

4. **Where were you born?** (Check one)

   ☐ US (1) ☐ Mexico* (2) ☐ Central America* (3) ☐ Other* (4) _____________

   * If Mexico, Central America or other, how many years have you been living in the US? ________

5. **How many (number) people live in your household?** (Including yourself) _________________

6. **Of the following people, check all who you live with:** (1) - yes; (0) - no

☐ Mom only ☐ Mother and Father ☐ Sister or Brother ☐ Other relative

☐ Dad only ☐ Parent and Step-Parent ☐ Grandparents

BOUNCE
to build healthy families

Acculturation (Child)

Name/ ID: ____________________ **Date: ________________________**

Instructions: Check the box which best answers the question for you. Please answer every question!

	Only Spanish (1)	Spanish Better than English (2)	Both Equally (3)	English Better than Spanish (4)	Only English (5)
1. What language(s) do you read and speak?	☐	☐	☐	☐	☐
2. What language(s) do your parents speak to you in?	☐	☐	☐	☐	☐
3. What language(s) do you usually speak at home?	☐	☐	☐	☐	☐
4. In which language(s) do you usually think?	☐	☐	☐	☐	☐
5. What language(s) do you usually speak with your friends?	☐	☐	☐	☐	☐
6. In what language(s) are the TV programs you usually watch?	☐	☐	☐	☐	☐
7. In what language(s) are the radio programs you usually listen?	☐	☐	☐	☐	☐
8. In what language(s) are the movies, TV, radio programs you want to watch or listen to?	☐	☐	☐	☐	☐
9. In what language(s) do your parents speak with their parents?	☐	☐	☐	☐	☐

	All Hispanic (1)	More Hispanic than Non-Hispanic (2)	Both Equally (3)	More Non-Hispanic than Hispanic (4)	All Non-Hispanic (5)
10. Your close friends are:	☐	☐	☐	☐	☐
11. You like going to parties at which people are:	☐	☐	☐	☐	☐
12. The people you visit or who visit you are:	☐	☐	☐	☐	☐

SASH-Y, Barona, A., & Miller, J. A. (1994).

BOUNCE
to build healthy families

McKnight Risk Factor Survey – Teasing

ID/Name: ___________________________ **Date: _____________________**

Instructions: There are no right or wrong answers. We just want to know what you think. Circle your response for each question. If you have problems with any of the questions, please raise your hand and we will help you.

PEER

1. In the past year, how often have girls/young women (including sisters) made fun of you because of your weight?

Never	A Little	Sometimes	A Lot	Always
1	2	3	4	5

2. In the past year, how often have you tried to change your weight so you would not be teased by boys/young men (including brothers)?

Never	A Little	Sometimes	A Lot	Always
1	2	3	4	5

3. In the past year, how often have boys/young men (including brothers) made fun of you because of your weight?

Never	A Little	Sometimes	A Lot	Always
1	2	3	4	5

4. In the past year, how often have you tried to change your weight so you would not be teased by girls/young women (including sisters)?

Never	A Little	Sometimes	A Lot	Always
1	2	3	4	5

5. If boys (including brothers) have teased you about your weight in the past year, how much has it changed the way you feel about yourself?

I have not been teased	Not At All	A Little	Pretty Much	A Lot	Totally
0	1	2	3	4	5

6. If girls (including sisters) have teased you about your weight in the past year, how much has it changed the way you feel about yourself?

I have not been teased	Not At All	A Little	Pretty Much	A Lot	Totally
0	1	2	3	4	5

7. In the past year, how much do you think your weight made <u>boys</u> not like you?

Not At All	A Little	Pretty Much	A Lot	Totally
1	2	3	4	5

8. In the past year, how much do you think your weight made other <u>girls</u> NOT like you?

Not At All	A Little	Pretty Much	A Lot	Totally
1	2	3	4	5

<u>ADULTS</u>

9. In the past year, how often has your father made a comment to you about your weight or your eating that made you feel bad?
(Remember that "father" is the adult man in your life who acts most like a father to you.)

I do not have contact with anyone that I think of as a "father"	Not At All	A Little	Pretty Much	A Lot	Totally
0	1	2	3	4	5

10. In the past year, how often has your mother made a comment to you about your weight or your eating that made you feel bad?
(Remember that "mother" is the adult in your life who acts like a mother to you.)

I do not have contact with anyone that I think of as a "mother"	Not At All	A Little	Pretty Much	A Lot	Totally
0	1	2	3	4	5

11. In the past year, how often has a teacher or coach made a comment to you about your weight that made you feel bad?

Never	A Little	Sometimes	A Lot	Always
1	2	3	4	5

BOUNCE
to build healthy families

McKnight Risk Factor Survey – Weight-Related Issues

ID/Name: ___________________________ **Date: _____________________**

Instructions: There are no right or wrong answers. We just want to know what you think. Circle your response for each question. If you have problems with any of the questions, please raise your hand and we will help you.

WEIGHT CONTROL BEHAVIORS

1. In the past month, how often have you tried to lose weight?

Never	A Little	Sometimes	A Lot	Always
1	2	3	4	5

2. In the past month, how often have you been on a diet to lose weight?

Never	A Little	Sometimes	A Lot	Always
1	2	3	4	5

3. In the past month, how often have you starved (not eaten) for a day or more to lose weight?

Never	A Little	Sometimes	A Lot	Always
1	2	3	4	5

4. In the past month, how often have you cut back on what you ate to lose weight?

Never	A Little	Sometimes	A Lot	Always
1	2	3	4	5

5. In the past month, how often have you skipped meals to lose weight?

Never	A Little	Sometimes	A Lot	Always
1	2	3	4	5

6. In the past month, how often have you exercised to lose weight?

Never	A Little	Sometimes	A Lot	Always
1	2	3	4	5

7. In the past month, how often have you eaten less sweets or fatty foods to lose weight?

Never	A Little	Sometimes	A Lot	Always
1	2	3	4	5

8. In the past month, how often have you kept eating and eating and felt like you could not stop?

Never	A Little	Sometimes	A Lot	Always
1	2	3	4	5

9. In the past month, how often did you eat a lot of food in a short amount of time when is was NOT a meal or a holiday?

Never	A Little	Sometimes	A Lot	Always
1	2	3	4	5

10. In the past month, how often have you taken laxatives or "water" pills to lose weight?

Never	A Little	Sometimes	A Lot	Always
1	2	3	4	5

11. In the past month, how often have you MADE yourself throw-up to lose weight?

Never	A Little	Sometimes	A Lot	Always
1	2	3	4	5

12. In the past month, how often have you taken diet pills to lose weight?

Never	A Little	Sometimes	A Lot	Always
1	2	3	4	5

13. In the past month, how often have you used teas or home remedies to lose weight?

Never	A Little	Sometimes	A Lot	Always
1	2	3	4	5

EMOTIONAL EATING

14. In the past month, how often did you eat less than usual when you were bored?

Never	A Little	Sometimes	A Lot	Always
1	2	3	4	5

15. In the past month, how often did you eat less than usual to try to feel better about yourself?

Never	A Little	Sometimes	A Lot	Always
1	2	3	4	5

16. In the past month, how often did you eat less than usual when you were upset?

Never	A Little	Sometimes	A Lot	Always
1	2	3	4	5

17. In the past month, how often did you eat more than usual when you were bored?

Never	A Little	Sometimes	A Lot	Always
1	2	3	4	5

18. In the past month, how often did you eat more than usual to try to feel better about yourself?

Never	A Little	Sometimes	A Lot	Always
1	2	3	4	5

19. In the past month, how often did you eat more than usual when you were upset?

Never	A Little	Sometimes	A Lot	Always
1	2	3	4	5

PARENT ENGLISH MEASURES

Clinical Measures (Parent)

BOUNCE to build healthy families

ID: ______________________________ **Date:** ____________________________

Race/Ethnicity (circle one): White Black/African American Hispanic Other

Gender (circle one): Female Male (Circle) PRE POST Other ______

"What is your height?": ______________ **"What is your weight?": ______________**

"What is your child's height?": ______________ **"What is your child's weight?": ______________**

Variable	Result		Average	Initials
**Today's Date** (mm/dd/yyyy)				
**DOB** (mm/dd/yyyy)				
**Age** (yrs)				
**Body Ht** (ft' in" i.e. 5' 2") (in and cm)	Trial 1<br>_______' _______"<br>in: _________<br>cm: _________	Trial 2<br>_______' _______"<br>in: _________<br>cm: _________	_______' _______"<br>in: _________<br>cm: _________	
**Body Wt.** (lbs. and kg)	Trial 1<br>lbs: _________<br>kg: _________	Trial 2	lbs.: _________<br>kg: _________	
**Body Fat** (%) (BIA)	Trial 1<br>_________%	Trial 2		
**Waist Circumference** (cm)	Trial 1<br>______________	Trial 2<br>______________	______________	
**Hip Circumference** (cm)	Trial 1<br>______________	Trial 2<br>______________	______________	
**Heart Rate** (bpm)	Trial 1<br>___________X2	Trial 2<br>___________X2	___________X2	
**Blood Pressure** (mmHg)	Trial 1<br>systolic: ________<br>diastolic: ________	Trial 2<br>systolic: ________<br>diastolic: ________	systolic: ________<br>diastolic: ________	
**1-mile Walk** (minutes)	Trial 1<br>1 mile minutes: ________<br>Pulse rate: ________<br>___________X2	N/A		
**BMI** (To be calculated after measurement)	BMI = $\frac{\text{Weight (kg)}}{(\text{Height(m)})^2}$ = ____________ =			
**Waist to Hip Ratio** (To be calculated after measurement)	Ratio = $\frac{\text{Waist circumference}}{\text{Hip circumference}}$ = ____________ =			

Date Entered: ______________________________ Initials: _______________

Date Double-checked: ________________________ Initials: _______________

Demographics & Technology (Parent)

ID/Name: _______________ Date: _________________

Instructions: Check the box or fill in your response for each question. Please answer every question.

1. When were you born? ______________ month __________________ day ________________ year

   Age: ___________ years

2. Where were you born? (Check one)

   ☐ US (1) ☐ Mexico (2) ☐ Central America (3) ☐ Other (7) ________________

2a. If Mexico, Central America or other, how many years have you been living in the US?_________

3. Check the box that best applies to you. Check **only** one.

   ☐ You were born in Mexico or another country. (1)

   ☐ You were born in the USA; either parent born in Mexico or another country. (2)

   ☐ You were born in the USA, both parents born in the USA and all grandparents born in Mexico or other country. (3)

   ☐ You and your parents were born in USA and at least one grandparent born in Mexico or other country with the remainder born in the USA. (4)

   ☐ You and your parents were born in the USA and all grandparents born in the USA. (5)

4. What is your ethnicity?

   ☐ Mexican American/Mexican (1)
   ☐ Central/South American (2)
   ☐ non-Hispanic Black (3)
   ☐ Other (4)_____________________

5a. What is the last grade you completed in school?

   ☐ Elementary – 6 (1)
   ☐ 7-8 (2)
   ☐ 9-12 (3)
   ☐ 1-2 years of college (4)
   ☐ 3-4 years of college (5)
   ☐ College Graduate or higher (6)
   ☐ Vocational/ Technical (7)

What is the last grade your spouse/partner completed in school?

   ☐ Elementary – 6 (1)
   ☐ 7-8 (2)
   ☐ 9-12 (3)
   ☐ 1-2 years of college (4)
   ☐ 3-4 years of college (5)
   ☐ College Graduate or higher (6)
   ☐ Vocational/ Technical (7)

5b. In what country? ____________________

In what country? ________________________

Acculturation (Parent)

ID/Name: ______________________________ **Date:** ______________________

Instructions: Check the box for each question which best answers each question for you. Please answer every question.

	Almost Never (1)	Sometimes (2)	Often (3)	Almost Always (4)
1. How often do you speak English?	☐	☐	☐	☐
2. How often do you speak in English with your friends?	☐	☐	☐	☐
3. How often do you think in English?	☐	☐	☐	☐
4. How often do you speak Spanish?	☐	☐	☐	☐
5. How often do you speak in Spanish with your friends?	☐	☐	☐	☐
6. How often do you think in Spanish?	☐	☐	☐	☐

	Very Poorly (1)	Poorly (2)	Well (3)	Very Well (4)
7. How well do you speak English?	☐	☐	☐	☐
8. How well do you read English?	☐	☐	☐	☐
9. How well do you understand television programs in English?	☐	☐	☐	☐
10. How well do you understand radio programs in English?	☐	☐	☐	☐
11. How well do you write in English?	☐	☐	☐	☐
12. How well do you understand music in English?	☐	☐	☐	☐
13. How well do you speak Spanish?	☐	☐	☐	☐
14. How well do you read in Spanish?	☐	☐	☐	☐
15. How well do you understand television programs in Spanish?	☐	☐	☐	☐

	Very Poorly<br><br>(1)	Poorly<br><br>(2)	Well<br><br>(3)	Very Well<br>(4)
16. How well do you understand radio programs in Spanish?	☐	☐	☐	☐
17. How well do you write in Spanish?	☐	☐	☐	☐
18. How well do you understand music in Spanish?	☐	☐	☐	☐

	Almost Never<br>(1)	Sometimes<br><br>(2)	Often<br><br>(3)	Almost Always<br>(4)
19. How often do you watch television programs in English?	☐	☐	☐	☐
20. How often do you listen to radio programs in English?	☐	☐	☐	☐
21. How often do you listen to music in English?	☐	☐	☐	☐
22. How often do you watch television programs in Spanish?	☐	☐	☐	☐
23. How often do you listen to radio programs in Spanish?	☐	☐	☐	☐
24. How often do you listen to music in Spanish?	☐	☐	☐	☐

BOUNCE
to build healthy families

Parenting for Eating and Physical Activity Survey (PEAS)

ID/Name:________________ **Date:**______________

How much do you agree or disagree with each statement?	Disagree	Slightly Disagree	Neutral	Slightly Agree	Agree
1. I offer sweets (candy, ice cream, cake) to my child as a reward for good behavior.	☐1	☐2	☐3	☐4	☐5
2. My child should always eat all the food on his/her plate.	☐1	☐2	☐3	☐4	☐5
3. I have to be especially careful to make sure my child eats enough.	☐1	☐2	☐3	☐4	☐5
4. If my child says "I'm not hungry," I try to get him/her to eat anyway.	☐1	☐2	☐3	☐4	☐5
5. If I don't regulate or guide my child's eating, he/she would eat much less than he/she should.	☐1	☐2	☐3	☐4	☐5
6. I limit the amount of soda my child drinks.	☐1	☐2	☐3	☐4	☐5
7. I limit the number of snacks my child eats.	☐1	☐2	☐3	☐4	☐5
8. I limit the amount of time my child watches TV or videos during the week (Mon-Fri)	☐1	☐2	☐3	☐4	☐5
9. I limit the amount of time my child watches TV or videos during the weekend (Sat/Sun)	☐1	☐2	☐3	☐4	☐5
10. I limit the amount of time my child plays video games (*like Game boy, Sega, Play station*) or is on the computer during the week (Mon-Fri)	☐1	☐2	☐3	☐4	☐5
11. I limit the amount of time my child plays video games (*like Game boy, Sega, Play station*) or is on the computer during the weekend (Sat/Sun)	☐1	☐2	☐3	☐4	☐5
12. I offer TV, videos, or video games to my child as a reward for good behavior.	☐1	☐2	☐3	☐4	☐5

How much do you keep track of the…?	Never	Rarely	Some times	Most of the time	Always
13. Sweet snacks (candy, ice cream, cake) that your child eats?	☐1	☐2	☐3	☐4	☐5
14. Salty snack foods (potato chips, tortilla chips) that your child eats?	☐1	☐2	☐3	☐4	☐5
15. High-fat foods that your child eats?	☐1	☐2	☐3	☐4	☐5
16. Amount of TV or videos your child is watching?	☐1	☐2	☐3	☐4	☐5
17. Exercise your child is getting?	☐1	☐2	☐3	☐4	☐5
18. Servings of fruits and vegetables your child is eating?	☐1	☐2	☐3	☐4	☐5

How often must your child ask permission before…?	Never	Rarely	Some times	Mostly	Always
19. Getting a snack	☐1	☐2	☐3	☐4	☐5
20. Drinking soda	☐1	☐2	☐3	☐4	☐5

How often do you…?	Never	Rarely	Some times	Mostly	Always
21. Praise your child for eating a healthy snack	☐1	☐2	☐3	☐4	☐5
22. Praise your child for being physically active	☐1	☐2	☐3	☐4	☐5

How often do you discipline your child for doing the following without your permission?	Never	Rarely	Some times	Mostly	Always
23. Watching TV or videos	☐1	☐2	☐3	☐4	☐5
24. Playing video games or the computer	☐1	☐2	☐3	☐4	☐5
25. Getting a snack	☐1	☐2	☐3	☐4	☐5
26. Drinking a soda	☐1	☐2	☐3	☐4	☐5
